# Maternal Plasma Phosphatidylcholine Fatty Acids and Atopy and Wheeze in the Offspring at Age of 6 Years

**DOI:** 10.1155/2012/474613

**Published:** 2012-09-25

**Authors:** Katharine C. Pike, Philip C. Calder, Hazel M. Inskip, Sian M. Robinson, Graham C. Roberts, Cyrus Cooper, Keith M. Godfrey, Jane S. A. Lucas

**Affiliations:** ^1^Clinical and Experimental Sciences Academic Unit, Faculty of Medicine, University of Southampton, Southampton S016 6YD, UK; ^2^NIHR Southampton Respiratory Biomedical Research Unit, University of Southampton and University Hospital Southampton NHS Foundation Trust, Southampton S016 6YD, UK; ^3^Human Development and Health Academic Unit, Faculty of Medicine, University of Southampton, Southampton S016 6YD, UK; ^4^NIHR Southampton Biomedical Research Centre, University of Southampton and University Hospital Southampton NHS Foundation Trust, Southampton S016 6YD, UK; ^5^Southampton Medical Research Council Lifecourse Epidemiology Unit, University of Southampton, Southampton S016 6YD, UK

## Abstract

Variation in exposure to polyunsaturated fatty acids (PUFAs) might influence the development of atopy, asthma, and wheeze. This study aimed to determine whether differences in PUFA concentrations in maternal plasma phosphatidylcholine are associated with the risk of childhood wheeze or atopy. For 865 term-born children, we measured phosphatidylcholine fatty acid composition in maternal plasma collected at 34 weeks' gestation. Wheezing was classified using questionnaires at 6, 12, 24, and 36 months and 6 years. At age of 6 years, the children underwent skin prick testing, fractional exhaled nitric oxide (FENO) measurement, and spirometry. Maternal *n*-6 fatty acids and the ratio of *n*-3 to *n*-6 fatty acids were not associated with childhood wheeze. However, higher maternal eicosapentaenoic acid, docosahexaenoic acid, and total *n*-3 fatty acids were associated with reduced risk of non-atopic persistent/late wheeze (RR 0.57, 0.67 and 0.69, resp. *P* = 0.01, 0.015, and 0.021, resp.). Maternal arachidonic acid was positively associated with FENO (*P* = 0.024). A higher ratio of linoleic acid to its unsaturated metabolic products was associated with reduced risk of skin sensitisation (RR 0.82, *P* = 0.013). These associations provide some support for the hypothesis that variation in exposure to *n*-6 and *n*-3 fatty acids during pregnancy influences the risk of childhood wheeze and atopy.

## 1. Introduction

Changes in dietary fat consumption have paralleled increases in childhood asthma and atopy in industrialised countries [1]. Consumption of oily fish, a source of long-chain *n*-3 polyunsaturated fatty acids (PUFAs), has declined and vegetable oils, a source of the *n*-6 PUFA linoleic acid (LA; 18:2*n*-6), have partly replaced animal fats [2–4]. As a result of these changes, long-chain *n*-3 PUFA and saturated fat intakes have decreased whilst LA intakes have increased [2–4]. Epidemiological data suggest that dietary patterns might influence allergic disease prevalence [1]. For example, reduced asthma prevalence has been reported in children who regularly eat fish [5, 6] and in the children of women who have high fish or *n*-3 PUFA intakes during pregnancy [7, 8]. Conversely, a high intake of vegetable oil-based spreads, a source of LA, is associated with an increased risk of asthma in children [6, 9]. These observations suggest opposing actions on *n*-6 and *n*-3 PUFAs on risk of asthma and allergy [10–12]. 

LA is converted to a longer chain more unsaturated *n*-6 PUFA arachidonic acid (AA; 20:4*n*-6) [13] which is the precursor of immunoregulatory eicosanoids like prostaglandin E_2_. The plant *n*-3 PUFA *α*-linolenic acid (ALA; 18:3*n*-3) is converted to longer chain more unsaturated *n*-3 PUFAs including eicosapentaenoic acid (EPA; 20:5*n*-3) and docosahexaenoic acid (DHA; 22:6*n*-3) [3, 13]. Conversion of both LA and ALA to their more unsaturated derivatives involves the same metabolic pathway [3, 13]. Thus a high exposure to LA impairs the conversion of ALA, so favouring synthesis of AA over EPA and DHA [13]. Therefore, the balance of *n*-3 and *n*-6 PUFAs is considered to be important [3, 13]. This balance might influence risk of atopy and asthma via effects upon prostaglandin E_2_ formation, with consequences for T cell and B cell responses and allergic sensitisation [10–12]. Like oily fish, fish oil is a source of EPA and DHA. Maternal fish oil supplementation in pregnancy has been shown to alter neonatal T-cell cytokine production [14] and to reduce asthma risk in the offspring [15]. However, supplementation of infants with fish oil seems not to reduce allergic sensitisation or asthma [16, 17], although studies of asthma-related outcomes in older children are equivocal [18, 19]. As allergic sensitisation occurs early in life, exposures at this time are most likely to influence immune development. Some studies have suggested that cord blood *n*-6 and *n*-3 PUFAs differ between infants according to personal or family history of atopy [20–23] and have been interpreted as evidence of disturbed fatty acid metabolism in atopic individuals [24]. However, studies of small “high-risk” populations are poorly generalisable and are often underpowered to assess clinical outcomes. More recently, associations between maternal or cord blood PUFA status and clinical outcomes have been sought prospectively in large birth cohorts. One such study found a positive association between the ratio of *n*-3 to *n*-6 PUFAs in maternal plasma phospholipids and eczema in early childhood [25]. In contrast, another study found inverse associations between the ratio of *n*-3 to *n*-6 PUFAs in umbilical cord blood red cells and both eczema and late-onset wheeze, a sign of asthma [26], although the findings were regarded as nonsignificant after adjusting for multiple comparisons. Thus, currently the relationship between early exposure to different PUFAs and later outcomes related to atopy and asthma is not clear and warrants further study. In particular, it remains unclear whether natural variation in maternal PUFA status influences childhood atopy or wheeze risk. Here, we examine the relationship between maternal PUFA status in late pregnancy and allergic disease in childhood. The primary hypothesis being investigated is that high *n*-6 PUFAs (individual and total), low *n*-3 PUFAs (individual and total), and a low ratio of *n*-3 to *n*-6 PUFAs at 34 weeks' gestation are associated with increased risk of childhood atopy and wheeze. The secondary hypothesis is that a higher ratio of precursor essential fatty acids to their unsaturated metabolic products, suggestive of decreased conversion of the plant-derived precursor PUFAs to their biologically active derivatives, is associated with increased childhood atopy and wheeze risk. Because of the mutual competition between LA and ALA metabolism, a high exposure to LA would be expected to increase the ratio of ALA to its derivatives. Conversely, a lower exposure to LA would be expected to decrease this ratio. 

## 2. Methods

### 2.1. Study Population

**Figure 1:**
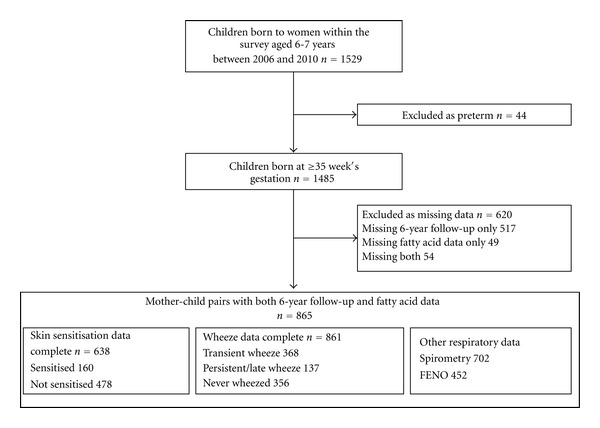
Study outline.

Participants were mothers and children in the Southampton Women's Survey [27]. During 1998–2002, 12,583 20–34-year-old women were recruited; those who became pregnant were followed up and their children visited at ages 6, 12, 24, and 36 months and 6 years. Infants born ≥ 35 weeks' gestation were studied to exclude abnormal lung development associated with prematurity. 1485 children born ≥ 35 weeks' gestation were aged 6-7 years during the study period 2006–2010; of these, 865 had both maternal PUFA measurements and 6-year follow-up data (Figure 1). This study was conducted according to the guidelines laid down in the Declaration of Helsinki and all procedures involving human subjects were approved by the Southampton and South West Hampshire Local Research Ethics Committee (276/97, 307/97, 089/99, and 06/Q1702/104). Written informed consent was obtained from all participant women for both their participation and that of their child. 

### 2.2. Maternal Plasma Phosphatidylcholine PUFA Composition

Venous blood was sampled at 34 weeks' gestation. Samples were centrifuged and plasma stored at −80° C. Phosphatidylcholine (PC) is the major phospholipid in plasma. PC fatty acid composition was determined by gas chromatography. Plasma lipids were extracted using chloroform/methanol (2 : 1) and PC separated by solid phase extraction (aminopropylsilica Bond-Elut cartridge, Varian Inc., CA). PC fatty acids were converted to methyl esters by heating with sulphuric acid containing 2% methanol. Fatty acid methyl esters (FAMEs) were extracted into hexane and concentrated by evaporation under nitrogen. FAMEs were separated by gas chromatography (Series 6890, Hewlett Packard, BPX 70 column SGE Europe Ltd.) and were identified by comparing retention times with those of authentic standards. Data are expressed as percentage concentration (g/100 g total fatty acids). Ten fatty acid exposures were calculated: total *n*-3 and *n*-6 PUFAs as percentage of total fatty acids and the ratio of total *n*-3: total *n*-6 PUFAs; percentages of the essential *n*-3 precursor ALA, the essential *n*-6 precursor LA, the *n*-3 products of ALA metabolism EPA and DHA, and the *n*-6 product of LA metabolism AA; the ratios of essential fatty acid precursors (i.e., LA or ALA) to their unsaturated products for both the *n*-3 and *n*-6 PUFA families (ALA/(20:4*n*-3 + EPA + 22:5*n*-3 + DHA) and LA/(18:3*n*-6 + 20:3*n*-6 + AA + 22:4*n*-6 + 22:5*n*-6)). For the latter, a high ratio is a proxy indicator of lower conversion efficiency of the essential fatty acid precursor to its unsaturated products. 

### 2.3. Atopy

Skin prick testing was used to identify immunologic sensitization to a range of common allergens; such sensitization is termed atopy. Skin prick testing was conducted in the children at age of 6 years in their homes using cat, dog, house dust mite, egg, milk, and grass and tree pollen allergens. Skin prick tests were considered valid if positive and negative control wheals were ≥3 mm and 0 mm, respectively. Atopy was defined as a wheal ≥3 mm to any allergen. 

### 2.4. Airway Inflammation

Upon attaining their sixth birthdays, children were invited to a clinical visit. Those attending attempted fractional exhaled nitric oxide (FENO) measurement, an indicator of the extent of airways inflammation. FENO was measured using a NIOX chemiluminescence analyser (Aerocrine, Sweden) according to the recommendations of European Respiratory Society-American Thoracic Society [28, 29]. Mean FENO was calculated, where possible, from three readings. 

### 2.5. Childhood Wheeze

Wheezing occurs when lower airways are narrow or constricted which may be due to asthma or to other lower respiratory conditions. Transient wheezing is common in young children, often due to the presence of an infection and usually stops altogether after about 3 years of age. Persistent wheezing is where wheezing continues beyond the preschool years; these children are more likely to have allergies and to develop asthma than those whose wheezing stops. Late-onset wheezing is where the wheeze does not develop until beyond the preschool years; again these children are more likely to have allergies and to develop asthma. Research nurses administered questions from the “International Study of Asthma and Allergies in Childhood” core questionnaire wheezing module [30] to the children's parents when the children were aged 6, 12, 24, and 36 months and 6 years. Mothers were asked whether their child had had ‘any episodes of chestiness associated with wheezing or whistling in his/her chest since they were last seen?' The results were combined to define

- transient wheeze: wheeze at 6, 12, 24, or 36 months but no wheeze at 6 years and no asthma treatment at 6 years;
- persistent wheeze: wheeze at 6, 12, 24, or 36 months, plus wheeze at 6 years or asthma treatment at 6 years;
- late-onset wheeze: no wheeze at 6, 12, 24, or 36 months, plus wheeze at 6 years or asthma treatment at 6 years.

The persistent and late-onset wheeze groups were combined as few children wheezing at 6 years did not wheeze before 36 months. Persistent/late wheeze was subclassified according atopic status established through skin prick testing.

### 2.6. Lung Function

Spirometry was performed at age 6 years in the children's homes according to the American Thoracic Society guidelines [31, 32]; to avoid discomfort nose clips were not used. Flow-volume loops were measured using a Koko spirometer with incentive software. Absolute forced expiratory volume at 1 second (FEV_1_) values were recorded without height standardisation because it was believed possible that any effect of maternal PUFA status upon childhood airway dimension, and hence wheeze risk might be mediated by an effect upon childhood height. 

### 2.7. Statistical Methods

Poisson regression with robust variance was used to model relative risk for binary outcomes. This approach is appropriate for common outcomes where odds ratios derived from logistic regression cannot be interpreted as relative risks [33]. As the transient and persistent/late wheeze phenotypes were mutually exclusive, children suffering one of these types of wheeze could not be regarded as at risk of the other. For this reason, relative risks were calculated by comparing children with transient or persistent/late wheeze to those who had never wheezed. Persistent/late wheeze with atopy is believed to be a separate phenotype to persistent/late wheeze without atopy differing not only in clinical presentation but likely also in aetiology. Relative risks for these phenotypes were calculated using nonatopic children who had never wheezed as the comparator group. Relationships between maternal PUFAs and continuous outcomes were explored using linear regression. An inverse square root transformation was used to normalise FENO data. For ease of interpretation, transformed FENO values were standardised and the sign switched so that high untransformed FENO values gave rise to high standardised scores. 

 The variables listed in Table 1 were considered potential confounders a priori except birth weight and gestational age as these factors may causally link maternal PUFA status and childhood outcomes. Potential confounders were tested for association with each respiratory outcome and models developed comprising all variables associated with each outcome; for this reason, different outcomes will have different confounders. Exposure variables were standardised so that each variable was on the same scale so that relative risks could be compared as change in risk per one standard deviation increment in each exposure variable. The assumption of linearity was confirmed using a quadratic term to exclude a nonlinear, or U-shaped, association. 

Interpretation of the analyses is complicated by the number of statistical tests made, and the conventional *P* value of 0.05 should be interpreted with caution. However, because the analyses were designed a priori to test specific hypotheses and not all the tests were independent, a Bonferroni correction would be over-conservative [34]. We therefore focused primarily on results with *P* values ≤0.025 and considered the consistency of the findings in our interpretation. Analyses were completed using Stata 11 (Stata Corp., College Station, TX). 

## 3. Results

### 3.1. Participants

PUFA data were available for 865 (94.6%) of the 914 mother-child pairs with 6 year follow-up data. The 865 mother-child pairs included in the study were broadly similar across a number of demographic characteristics to those missing either PUFA or follow-up data (Table 1). Study mothers were, however, slightly older, less likely to smoke in pregnancy and of higher education and social class, whilst study children were less likely to be exposed to tobacco smoke in infancy and more likely to have been breast fed than those without follow-up or fatty acid data (Table 1).

Sufficient questionnaire data were available to classify 856 children according to wheeze status; of these 635 children consented to skin prick testing and had valid results. 160 (25.1%) study children were classified as atopic; 368 (42.7%) had transient and 137 (15.9%) persistent/late wheeze. 94 children in the persistent/late group were characterized according to atopic status, of these 50.0% were atopic and 50.0% nonatopic (Table 2). These data are comparable to other cohorts in the United Kingdom [35, 36]. The median percentage PUFA concentrations values (Table 3) were comparable to published data from a similar gestation [37]. Acceptable FEV_1_ and FENO measurements were available from 702 and 452 children, respectively.

### 3.2. Maternal Late-Pregnancy PUFAs and Childhood Wheeze

Total maternal *n*-3 and *n*-6 PUFA percentages and the ratio of *n*-3 to *n*-6 PUFAs were not associated with any childhood wheeze phenotype (Table 4). The ratio of the *n*-3 fatty acid precursor (ALA) to its unsaturated metabolic products had weak positive associations with transient wheeze (adjusted RR 1.08, *P* = 0.03) and with persistent/late wheeze risk (adjusted RR 1.14, *P* = 0.03) (Table 4). The latter association was significant in nonatopic children only (adjusted RR 1.46, *P* = 0.010) (Table 5). A similar weak positive association was found between this ratio and transient wheeze (adjusted RR 1.08, *P* = 0.03) (Table 4). Significant inverse associations were found between total *n*-3 PUFAs, DHA and EPA, and nonatopic persistent/late wheeze (adjusted RR 0.69, *P* = 0.021; adjusted RR 0.67, *P* = 0.015; adjusted RR 0.57, *P* = 0.014) (Table 5). EPA, DHA, and total *n*-3 PUFAs had weak inverse associations with atopic persistent/late wheeze (adjusted RR 0.65, *P* = 0.04; adjusted RR 0.74, *P* = 0.05; adjusted RR 0.72, *P* = 0.03) (Table 5). A significant inverse association was found between maternal AA and nonatopic persistent/late wheeze (adjusted RR 0.76, *P* = 0.023). There was no evidence for a significant association between total *n*-6 PUFAs and any wheeze phenotype, and the ratio of the *n*-6 fatty acid precursor (LA) to its unsaturated metabolic products had no significant associations with any wheeze outcome.

### 3.3. Maternal Late-Pregnancy PUFAs and Childhood Atopy

A higher ratio of the *n*-6 fatty acid precursor (LA) to its unsaturated metabolic products was associated with a decreased risk of childhood skin sensitisation (adjusted RR 0.82, *P* = 0.013) (Table 6). The *n*-6 fatty acid AA was not significantly associated with skin sensitisation and no significant associations were found for any other PUFA or ratio of PUFAs examined. 

### 3.4. Maternal Late-Pregnancy PUFAs and Childhood FENO

No associations were found between total *n*-3 or *n*-6 PUFAs or the ratio of *n*-3 to *n*-6 PUFAs and FENO (Table 6). However, maternal AA and FENO were positively associated (*P* = 0.024). 

### 3.5. Maternal Late-Pregnancy PUFAs and Childhood FEV_1_

None the PUFAs, nor any of the fatty acid ratios, examined was significantly associated with childhood FEV_1_ (Table 7). FEV_1_ was uncorrected for height due to concerns that height might mediate any association between maternal fatty acids and airway geometry; however, including child's height in the multivariate analysis did not alter the findings (results not shown).

## 4. Discussion

In this study we found some limited support for our primary hypothesis linking relatively low levels of maternal *n*-3 compared to *n*-6 PUFAs with later wheeze and atopy. Higher maternal EPA, DHA, and total *n*-3 PUFAs at 34 weeks' gestation were associated with a lower risk of persistent/late wheeze at age of 6 years. This association was only significant in nonatopic children, and there was no association between total *n*-3 PUFAs, total *n*-6 PUFAs or the ratio of *n*-3 to *n*-6 PUFAs, and childhood atopy as measured by skin sensitisation. However, maternal AA, a metabolic product of the essential *n*-6 precursor LA, was significantly positively associated with FENO, a marker of airways inflammation. Associations may reflect a direct action of intrauterine PUFA supply upon fetal development. Alternatively associations may occur as a consequence of dietary patterns or metabolic disturbance shared by mothers and their children. Little support was found for the secondary hypothesis, although the ratio of ALA to its unsaturated metabolic products was positively associated with persistent/late wheeze in nonatopic children and the ratio of LA to its unsaturated metabolic products was inversely associated with skin sensitisation at 6 years. However, these findings cannot easily be interpreted as evidence for decreased precursor conversion in atopic disease. 

The inverse associations between total maternal EPA, DHA, and total *n*-3 PUFAs and childhood persistent/late wheeze in the group as a whole, but particularly in those without atopy, are consistent with the idea that highly unsaturated *n*-3 fatty acids might protect against childhood wheeze, as suggested by Black and Sharpe [10]. However, only the associations with nonatopic persistent/late wheeze were significant, and no significant association was found between maternal *n*-3 PUFAs and skin sensitisation or atopic persistent/late wheeze. There was no evidence that higher maternal *n*-6 PUFAs increased the offspring's risk of wheeze or skin sensitisation. Whilst these results do not support competing effects of *n*-3 and *n*-6 PUFAs upon allergic skin sensitisation or the original Black and Sharp hypothesis [10], the positive association between maternal AA concentration and FENO suggests that eosinophilic airways inflammation is higher in the children of mothers with high AA status. 

 A previous study found no association between maternal or umbilical cord AA and atopy, although the study included less than 300 mother-child pairs and did not consider objective measures of atopy such as skin sensitization [38]. The weak inverse association between AA and persistent/late wheeze in nonatopic children found in the current study, however, contradicts the hypothesised association between high levels of *n*-6 PUFAs and asthma. Although this finding was unexpected and may have occurred by chance, inverse associations have previously been found between AA and risk of atopy in adults [24, 39] and in infants [20, 22]. This finding is also comparable to an inverse association found previously between the maternal ratio of *n*-6 to *n*-3 long-chain PUFAs and childhood eczema [25]. 

 The ratio of ALA to its unsaturated metabolic products was significantly positively associated with nonatopic persistent/late wheeze and weakly with transient wheeze. These results are compatible with the suggestion of altered fatty acid metabolism in mothers of children who later develop allergic disease [21], and also with genetic data linking polymorphisms in the gene clusters coding for fatty acid desaturases and the prevalence of allergic rhinitis and atopic eczema [40]. However, given that no associations were found between this ratio and skin sensitisation or persistent/late wheeze with atopy, there was no evidence that inefficiency in ALA metabolism is associated with childhood wheeze as a consequence of allergic sensitisation. However, the inverse association between the ratio of LA to its unsaturated metabolic products and skin sensitisation might indicate altered LA metabolism in the mothers of children who later develop atopy compared to mothers of nonatopic children. The direction of this association does not appear to support a link between decreased fatty acid desaturase activity and childhood atopy, although definitive assertions about flux through pathways cannot be made without stable isotope studies. 

The current study has some limitations. Although a priori hypotheses were examined, due to the number of associations tested, unwarranted biological significance may be attributed to false positive results. Response bias cannot be excluded as the entire original cohort was not followed up; this would only invalidate the study's conclusions, however, if the relationship between maternal PUFA status and childhood outcomes differed between study participants and those with missing data. A broad range of potential confounders were considered but residual confounding cannot be excluded. We were unable to correct for maternal PUFA status earlier in pregnancy, for breast milk fatty acid composition, or for the children's PUFA intake and status in postnatal life, which are all likely to be factors contributing to immune development and allergy risk in childhood. It is also unclear whether sufficient variation in exposure exists within this population to meaningfully affect outcomes; less than 1% of the mothers in this study reported an intake of at least one fatty fish meal per week, the intake providing the *n*-3 PUFA dose administered in a successful fish oil intervention trial [15]. Epidemiological studies are further complicated by the multiple asthma phenotypes thought to exist, as these may show different patterns of association with early life influences. Heterogeneity within study outcomes is likely, and this may decrease the study's sensitivity. A final limitation is that we measured the fatty acid composition of maternal plasma PC, the major phospholipid in the bloodstream. Although this has the advantage that plasma PC fatty acids are influenced by dietary intakes and that the context of the Black and Sharp hypothesis is an effect of maternal diet on later risk of allergy and asthma [10], plasma phospholipids are not the principal vehicle for supply of fatty acids from the maternal circulation to the fetus [41]. For this reason, the fatty acid composition of maternal plasma triacylglycerols and nonesterified fatty acids would also be useful to measure, although the PUFAs in the former correlate strongly with those in plasma PC, as would the fatty acid composition of cord blood plasma lipids. 

 In summary, measurement of maternal PUFAs in a large population-based cohort enabled us to investigate hypotheses linking PUFA exposure during late-pregnancy to childhood wheeze and atopy. An inverse association was found for maternal EPA, DHA, and total *n*-3 PUFAs and the relative risk of persistent/late nonatopic wheeze whilst high maternal AA was associated with increased FENO, a marker of airways inflammation. Together with a number of weaker associations, albeit with small effect sizes, these results provide some support for a protective link between the maternal *n*-3 PUFAs and wheeze and atopy in childhood. Support for the suggested role of *n*-6 PUFAs was minimal.

## Figures and Tables

**Table 1:** Comparison of SWS mother-child pairs with complete data with those lacking either maternal fatty acid or 6-year follow-up data but born in the same time period.

	Mother-child pairs in analysis (*n* = 865)	Mother-child pairs with missing data (*n* = 620)	*P*
Maternal characteristics			

Age at child's birth (mean (SD))	30.4 (3.8)	29.6 (3.8)	0.0003
Primiparous (*n* (%))			
No	463 (53.5)	368 (59.4)	0.03
Yes	402 (46.5)	252 (40.6)	
Education attainment (*n* (%))*			<0.001
None	14 (1.6)	35 (5.7)
GCSE D–G	84 (9.7)	67 (10.8)
GCSE A*–C	248 (28.7)	179 (28.9)
A Level	251 (29.1)	184 (29.7)
HND	64 (7.4)	40 (6.5)
University degree	203 (23.5)	114 (18.4)
Parents' social class (*n* (%))^†^			0.01
I	91 (10.6)	54 (11.7)
II	427 (49.9)	199 (43.3)
III Nonmanual	234 (27.4)	123 (26.7)
III Manual	67 (7.8)	54 (11.7)
IV	34 (4.0)	23 (5.0)
V	2 (0.2)	7 (1.5)
Smoked in pregnancy (*n* (%))			<0.001
No	728 (85.5)	464 (78.0)
Yes	123 (14.5)	131 (22.0)
Maternal asthma (*n* (%))			0.2
No	676 (78.8)	465 (76.1)
Yes	182 (21.2)	146 (23.9)
Maternal childhood eczema (*n* (%))			0.7
No	706 (82.4)	498 (81.5)
Yes	151 (17.6)	113 (18.5)
Maternal rhinitis (*n* (%))			0.3
No	497 (57.9)	369 (60.4)
Yes	361 (42.1)	242 (39.6)
Prepregnancy BMI, kg/m^2^ (median, IQR)	24.3 (22.0–27.6)	24.0 (21.9–27.3)	0.5
Total energy intake, kcal (median, IQR)	2301 (1919–2748)	2354 (1958–2779)	0.08

Paternal characteristics			

Paternal asthma (*n* (%))			0.5
No	699 (82.1)	484 (80.8)
Yes	152 (17.9)	115 (19.2)
Paternal childhood eczema (*n* (%))			0.9
No	743 (88.2)	527 (88.1)
Yes	99 (11.8)	71 (11.9)
Paternal rhinitis (*n* (%))			0.5
No	599 (66.3)	399 (66.5)
Yes	284 (33.7)	201 (33.5)

Child's characteristics			

Gender (*n* (%))			0.5
Male	447 (51.7)	331 (53.6)
Female	418 (48.3)	287 (46.4)
Birth weight, kg (mean (SD))	3.49 (0.50)	3.46 (0.50)	0.4
Gestational age, weeks (mean (SD))	39.9 (1.4)	39.9 (1.5)	0.6
Months of breastfeeding (*n* (%))			<0.001
None	133 (15.4)	130 (23.5)
<1	170 (19.7)	113 (20.4)
1–3	153 (17.7)	119 (21.5)
4–6	153 (17.7)	70 (12.6)
7–11	143 (16.6)	81 (14.6)
12 or more	111 (12.9)	41 (7.4)
Age of introduction solid food, weeks (median (IQR))	17.0 (15–18)	17 (15–18)	0.3
Mother smoking during child's infancy (*n* (%))			0.003
No	712 (83.0)	434 (76.7)
Yes	146 (17.0)	132 (23.3)
Cats/dogs in home during child's infancy (*n* (%))			0.5
No	438 (50.8)	290 (52.8)
Yes	424 (49.2)	259 (47.2)

Numbers do not always add to the full column totals due to missing data

Binary outcomes were compared by *χ* ^2^ test, categorical outcomes by a *χ* ^2^ test for trend, and continuous variables using *t*-tests, after transformation where appropriate, or a ranksum test.

*GCSE General certificate of secondary education, high school education (to age 16) graded from G (low) to A* (high), A level Advanced-level high school education (to age 18), HND Higher national diploma higher education qualification of slightly lower level than that of a university degree.

^†^Social class graded from V (low) to (I) high according to occupation.

**Table 2:** Distribution of participants between outcome groups.

Outcome	*N*	%
Never wheezed	356	41.3
Transient wheeze	368	42.7
Persistent/late wheeze	137	15.9
Persistent/late wheeze with atopy	47	7.4
Persistent/late wheeze without atopy	47	7.4
Skin sensitisation	160	25.1

Wheeze classification possible for 861 children, skin sensitisation data available for 638, and both wheeze and skin sensitisation data available for 635.

**Table 3:** Fatty acid composition of maternal plasma PC measured at 34 weeks' gestation.

Fatty acid exposure	Median	25th and 75th percentiles
Total *n*-3 PUFAs %	5.01	4.34, 5.84
ALA %	0.29	0.22, 0.36
DHA %	3.78	3.25, 4.41
EPA %	0.36	0.27, 0.48
Total *n*-6 PUFAs %	35.4	34.0, 36.8
LA %	23.1	21.6, 24.7
AA %	7.67	6.79, 8.54
Total *n*-3 : *n*-6 PUFAs	0.14	0.12, 0.17
Ratio of ALA to its unsaturated metabolic products	0.06	0.04, 0.08
Ratio of LA to its unsaturated metabolic products	1.87	1.66, 2.14

*N* = 865.

ALA: alpha-linolenic acid; DHA: docosahexaenoic acid; EPA: eicosapentaenoic acid; LA: linoleic acid; AA: arachidonic acid.

**Table 4:** Relative risks (RR) for the association between standardised values (*z*-scores) of maternal plasma PC fatty acids and wheeze phenotypes.

	Transient wheeze						Persistent/late wheeze					
Maternal exposure	Unadjusted analysis *n* = 724			Adjusted* analysis *n* = 493			Unadjusted analysis *n* = 493			Adjusted^†^ analysis *n* = 479		
	RR	(95% CI)	*P*	RR	(95% CI)	*P*	RR	(95% CI)	*P*	RR	(95% CI)	*P*
Total *n*-3 PUFAs %	0.94	(0.88, 1.02)	0.14	0.97	(0.90, 1.04)	0.40	0.85	(0.73, 0.99)	0.03	0.93	(0.79, 1.11)	0.44
ALA %	1.06	(0.98, 1.14)	0.13	1.06	(0.98, 1.14)	0.15	1.15	(1.01, 1.31)	0.04	1.09	(0.96, 1.24)	0.17
DHA %	0.92	(0.85, 1.00)	0.04	0.95	(0.88, 1.03)	0.19	0.81	(0.70, 0.95)	0.008	0.91	(0.76, 1.08)	0.28
EPA %	0.97	(0.90, 1.05)	0.51	0.98	(0.92, 1.06)	0.64	0.88	(0.72, 1.08)	0.22	0.91	(0.73, 1.14)	0.42
Total *n*-6 PUFAs %	0.98	(0.91, 1.05)	0.58	0.97	(0.91, 1.04)	0.37	1.05	(0.91, 1.21)	0.52	0.99	(0.85, 1.16)	0.92
LA %	1.01	(0.94, 1.08)	0.84	1.00	(0.94, 1.08)	0.89	1.05	(0.91, 1.21)	0.49	1.04	(0.90, 1.21)	0.60
AA %	0.95	(0.89, 1.02)	0.19	0.94	(0.88, 1.02)	0.13	0.91	(0.79, 1.04)	0.16	0.88	(0.76, 1.02)	0.09
Total *n*-3 : *n*-6 PUFAs ratio	0.96	(0.89, 1.04)	0.29	0.98	(0.92, 1.05)	0.63	0.85	(0.73, 0.99)	0.04	0.94	(0.79, 1.12)	0.49
ALA: unsaturated metabolic products	1.09	(1.02, 1.17)	0.012	1.08	(1.01, 1.16)	0.03	1.21	(1.07, 1.36)	0.002	1.14	(1.01, 1.28)	0.03
LA: unsaturated metabolic products	1.01	(0.94, 1.09)	0.72	1.02	(0.95, 1.09)	0.61	1.02	(0.90, 1.16)	0.73	1.06	(0.93, 1.21)	0.42

Children with transient and persistent/late wheeze were compared to children who had never wheezed. Unadjusted and adjusted analyses for each maternal variable are presented.

RR: relative risk; ALA: alpha-linolenic acid; DHA: docosahexaenoic acid; EPA: eicosapentaenoic acid; LA: linoleic acid; AA: arachidonic acid. RR derived from Poisson regression.

*Adjusted for maternal asthma and rhinitis, parity, child's sex, and maternal educational attainment.

^†^Adjusted for maternal asthma and rhinitis, paternal asthma, maternal smoking in pregnancy, child's sex, and maternal educational attainment.

**Table 5:** Relative risks (RR) for the association between standardised values (*z*-scores) of maternal plasma PC fatty acids and persistent/late wheeze with and without atopy.

	Persistent/late wheeze with atopy						Persistent/late wheeze without atopy					
Maternal exposure	Unadjusted analysis *n* = 260			Adjusted* analysis *n* = 224			Unadjusted analysis *n* = 260			Adjusted^†^ analysis *n* = 254		
	RR	(95% CI)	*P*	RR	(95% CI)	*P*	RR	(95% CI)	*P*	RR	(95% CI)	*P*
Total *n*-3 PUFAs %	0.8	(0.62, 1.02)	0.07	0.72	(0.54, 0.96)	0.03	0.62	(0.46, 0.84)	0.002	0.69	(0.51, 0.95)	0.021
ALA %	1.07	(0.80, 1.42)	0.65	0.91	(0.70, 1.19)	0.49	1.11	(0.84, 1.49)	0.46	1.17	(0.87, 1.58)	0.30
DHA %	0.79	(0.59, 1.05)	0.11	0.74	(0.55, 1.00)	0.05	0.59	(0.44, 0.81)	0.0009	0.67	(0.49, 0.93)	0.015
EPA %	0.74	(0.54, 1.01)	0.06	0.65	(0.43, 0.98)	0.04	0.61	(0.41, 0.92)	0.017	0.57	(0.37, 0.89)	0.014
Total *n*-6 PUFAs %	1.12	(0.88, 1.43)	0.35	1.05	(0.79, 1.39)	0.75	1.12	(0.86, 1.45)	0.40	1.00	(0.76, 1.30)	0.97
LA %	0.99	(0.76, 1.29)	0.94	0.88	(0.65, 1.20)	0.42	1.22	(0.92, 1.61)	0.18	1.17	(0.88, 1.54)	0.28
AA %	0.99	(0.78, 1.24)	0.90	1.06	(0.82, 1.36)	0.66	0.82	(0.65, 1.03)	0.08	0.76	(0.60, 0.96)	0.023
Total *n*-3 : *n*-6 PUFAs ratio	0.78	(0.60, 1.00)	0.05	0.73	(0.54, 0.99)	0.04	0.64	(0.46, 0.87)	0.005	0.73	(0.53, 0.99)	0.05
ALA: unsaturated metabolic products	1.22	(0.94, 1.59)	0.14	1.07	(0.85, 1.34)	0.58	1.43	(1.12, 1.82)	0.005	1.46	(1.10, 1.95)	0.010
LA: unsaturated metabolic products	0.88	(0.69, 1.12)	0.29	0.78	(0.59, 1.04)	0.09	1.14	(0.93, 1.40)	0.20	1.19	(0.97, 1.48)	0.10

Children with persistent/late wheeze with and without atopy were compared to children without atopy who had never wheezed. Unadjusted and adjusted analyses for each maternal variable are presented.

RR: relative risk; ALA: alpha-linolenic acid; DHA: docosahexaenoic acid; EPA: eicosapentaenoic acid; LA: linoleic acid; AA: arachidonic acid. RR derived from Poisson regression.

*Adjusted for maternal asthma and atopy, paternal asthma, child's sex, and dogs/cats in the home during the child's infancy.

^†^Adjusted for maternal asthma, paternal asthma, maternal smoking during pregnancy, and dogs/cats in the home during the child's infancy.

**Table 6:** Relative risks (RR) for the association between standardised values (*z*-scores) of maternal plasma PC fatty acids and atopy and regression coefficients for the association between standardised values (*z*-scores) of maternal plasma PC fatty acids and airways inflammation.

	Skin sensitisation						Fractional exhaled nitric oxide					
Maternal exposure	Unadjusted analysis *n* = 638			Adjusted* analysis *n* = 545			Unadjusted analysis *n* = 452			Adjusted^†^ analysis *n* = 435		
	RR	(95% CI)	*P*	RR	(95% CI)	*P*	Beta	(95% CI)	*P*	Beta	(95% CI)	*P*
Total *n*-3 PUFAs %	1.09	(0.97, 1.23)	0.16	0.99	(0.87, 1.13)	0.87	0.056	(−0.037, 0.148)	0.24	0.042	(−0.052, 0.135)	0.38
ALA %	0.95	(0.83, 1.09)	0.49	0.99	(0.86, 1.14)	0.90	−0.041	(−0.135, 0.053)	0.40	−0.05	(−0.144, 0.044)	0.29
DHA %	1.11	(0.98, 1.26)	0.10	1.00	(0.87, 1.14)	0.97	0.108	(0.016, 0.201)	0.021	0.095	(0.002, 0.189)	0.05
EPA %	1.01	(0.90, 1.13)	0.91	0.94	(0.83, 1.06)	0.29	−0.012	(−0.109, 0.086)	0.82	−0.022	(−0.119, 0.074)	0.65
Total *n*-6 PUFAs %	0.92	(0.81, 1.05)	0.22	0.94	(0.83, 1.08)	0.40	−0.014	(−0.108, 0.079)	0.76	−0.011	(−0.105, 0.083)	0.83
LA %	0.87	(0.76, 0.99)	0.03	0.86	(0.75, 0.99)	0.04	−0.058	(−0.151, 0.036)	0.23	−0.066	(−0.160, 0.028)	0.17
AA %	1.08	(0.95, 1.22)	0.26	1.10	(0.96, 1.26)	0.17	0.096	(0.001, 0.191)	0.05	0.108	(0.014, 0.201)	0.024
Total *n*-3 : *n*-6 PUFAs ratio	1.09	(0.98, 1.22)	0.13	1.00	(0.88, 1.14)	0.98	0.05	(−0.042, 0.141)	0.29	0.039	(−0.053, 0.131)	0.41
ALA: unsaturated metabolic products	0.93	(0.80, 1.08)	0.32	1.02	(0.87, 1.20)	0.83	−0.08	(−0.172, 0.013)	0.09	−0.08	(−0.174, 0.013)	0.09
LA: unsaturated metabolic products	0.85	(0.73, 0.98)	0.025	0.82	(0.70, 0.96)	0.013	−0.071	(−0.166, 0.024)	0.14	−0.086	(−0.180, 0.008)	0.07

Children with skin sensitisation were compared to those without skin sensitisation. The association with FENO is expressed as change in FENO per standard deviation change in percentage fatty acid composition. Unadjusted and adjusted analyses for each maternal variable are presented.

RR: relative risk; ALA: alpha-linolenic acid; DHA: docosahexaenoic acid; EPA: eicosapentaenoic acid; LA: linoleic acid; AA: arachidonic acid. RR derived from Poisson regression, Beta derived from linear regression.

*Adjusted for maternal asthma, atopy and parity, paternal rhinitis, parental social class, child's sex and age, and dogs/cats in the home during the child's infancy.

^†^Adjusted for child's age, maternal asthma, and paternal rhinitis.

**Table 7:** Regression coefficients for the association between standardised values (*z*-scores) of maternal plasma PC fatty acids and FEV_1_ at 6 years of age.

	Change in FEV_1_ (L/s) per SD change of each fatty acid exposure variable					
Maternal exposure	Unadjusted analysis *n* = 702			Adjusted* analysis *n* = 702		
	Beta	(95% CI)	*P*	Beta	(95% CI)	*P*
Total *n*-3 PUFAs %	0.013	(−0.001, 0.028)	0.07	0.010	(−0.004, 0.024)	0.15
ALA %	−0.012	(−0.026, 0.003)	0.11	−0.009	(−0.024, 0.005)	0.18
DHA %	0.011	(−0.003, 0.026)	0.12	0.008	(−0.006, 0.022)	0.28
EPA %	0.016	(0.001, 0.030)	0.03	0.013	(−0.001, 0.027)	0.07
Total *n*-6 PUFAs %	0.000	(−0.015, 0.014)	0.96	0.000	(−0.014, 0.014)	0.99
LA %	−0.004	(−0.019, 0.011)	0.59	−0.005	(−0.019, 0.009)	0.50
AA %	−0.001	(−0.016, 0.013)	0.84	0.000	(−0.014, 0.015)	0.99
Total *n*-3 : *n*-6 PUFAs ratio	0.013	(−0.001, 0.027)	0.08	0.010	(−0.004, 0.024)	0.16
ALA: unsaturated metabolic products	−0.016	(−0.031, −0.002)	0.03	−0.011	(−0.026, 0.003)	0.12
LA: unsaturated metabolic products	−0.007	(−0.022, 0.008)	0.36	−0.008	(−0.023, 0.006)	0.25

Unadjusted and adjusted analyses for each maternal variable are presented.

RR: relative risk; ALA: alpha-linolenic acid; DHA: docosahexaenoic acid; EPA: eicosapentaenoic acid; LA: linoleic acid; AA: arachidonic acid. Beta derived from linear regression.

*Adjusted for parity and child's sex and age.
